# Mediastinal, retroperitoneal, and subcutaneous emphysema due to sigmoid colon penetration: A case report and literature review

**DOI:** 10.1016/j.ijscr.2019.02.003

**Published:** 2019-02-10

**Authors:** Tomohiro Muronoi, Akihiko Kidani, Eiji Hira, Kayo Takeda, Shunsuke Kuramoto, Kazuyuki Oka, Yoshihide Shimojo, Hiroaki Watanabe

**Affiliations:** Department of Acute Care Surgery, Shimane University, Faculty of Medicine, Shimane, Japan

**Keywords:** Mediastinal emphysema, Pneumomediastinum, Retroperitoneal emphysema, Subcutaneous emphysema, Colonic perforation, Diverticulitis

## Abstract

**Introduction:**

Mediastinal and subcutaneous emphysema usually result from spontaneous rupture of the alveolar wall. We present an extremely rare case of massive mediastinal, retroperitoneal, and subcutaneous emphysema due to the penetration of the colon into the mesentery.

**Presentation of case:**

A 57-year-old man presented to our institution with a history of chest pain. The patient’s medical history included malignant rheumatoid arthritis during the use of steroids and an immunosuppressive agent. The patient had no signs of peritoneal irritation or abdominal pain. A chest radiography revealed subcutaneous emphysema of the neck, mediastinal emphysema, as well as subdiaphragmatic free air. Computed tomography showed extensive retroperitoneal, mediastinal, and mesenteric emphysema of the sigmoid colon without pneumothorax. Diagnostic laparoscopy was performed and revealed perforation into the sigmoid mesentery. Segmental resection of the sigmoid colon and end-colostomy were performed. The diverticulum was communicating with the outside of the mesentery via the mesentery. The mediastinal emphysema disappeared a few days after the surgery.

**Discussion:**

Colonic perforation generally results in free perforation. Colonic gas may spread via various anatomical pathways when perforation of the colon occurs in the retroperitoneum; thus, diverse atypical clinical symptoms may be present. Signs of peritoneal irritation can be hidden in cases of retroperitoneal colonic perforation. The atypical manifestation of a retroperitoneal colonic perforation can cause difficulties in making a diagnosis.

**Conclusions:**

Massive mediastinal and retroperitoneum emphysema are rare signs of colonic perforation. Emergency laparotomy should be considered in colonic penetration of the diverticulitis where the emphysema expands to the mediastinum extensively.

## Highlights

• Massive mediastinal emphysema is rare signs of colon perforation.
• The diagnosis may be delayed due to the absence of peritoneal irritation.
• Laparotomy should be considered in case of colonic penetration with the mediastinum emphysema.

## 1 Introduction

Mediastinal and subcutaneous emphysema usually result from spontaneous rupture of the alveolar wall [1]. These symptoms, following colonic perforation, are unusual, and massive mediastinal emphysema is extremely rare without a preceding endoscopic procedure [[2], [3], [4], [5]]. Most symptoms of colonic perforation are due to abscesses or peritonitis [6]. However, it is difficult to diagnose retroperitoneal penetration of the colon and rectum because of the lack of typical symptoms [7,8]. Furthermore, delayed diagnosis and treatment could result in high morbidity and mortality [6]. We present a rare case of colonic penetration with massive retroperitoneal, mediastinal, and subcutaneous emphysema. This report has been made according to the SCARE criteria [9].

## 2 Presentation of case

**Fig. 1:**
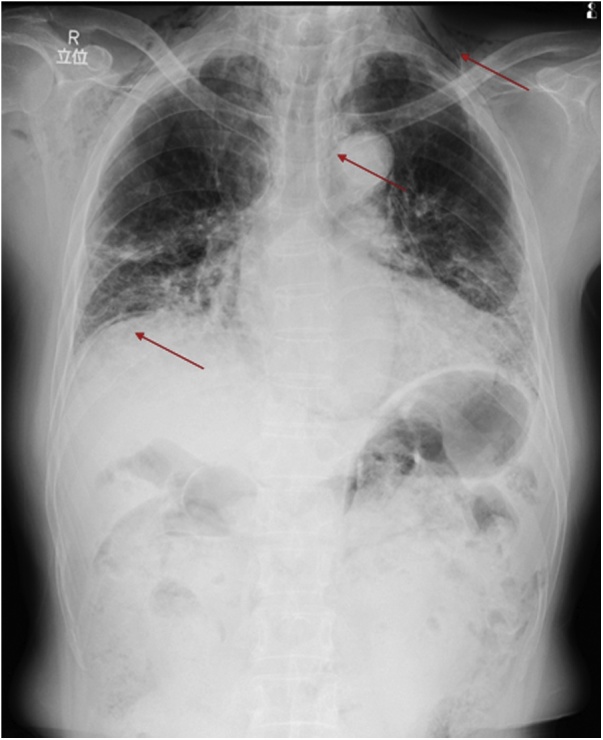
Chest radiography showing subcutaneous emphysema of the neck, mediastinal emphysema, and subdiaphragmatic free air (red arrows).

**Fig. 2:**
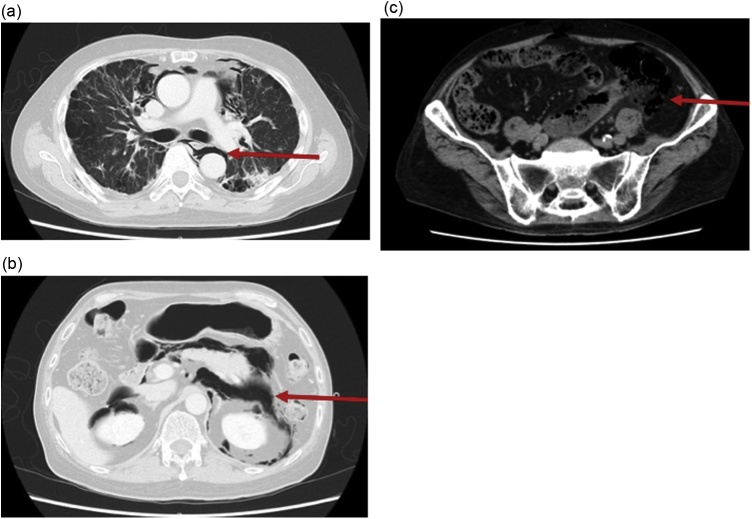
Selected images from a computed tomography (CT) scan of the chest, abdomen, and pelvis (a, b, and c: axial images; d: reformatted coronal images). CT demonstrated perforation of the sigmoid colon into the mesentery (c, red arrows). Using lung windowing, gas was found extensively, from the retroperitoneum to the mediastinum (a, b, and d, red arrows).

**Fig. 3:**
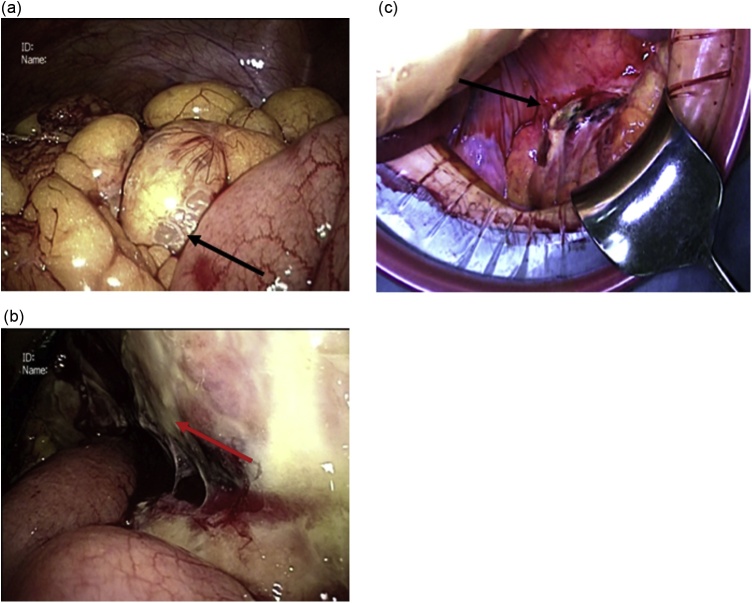
Intraoperative image showing intestinal emphysema (a) and necrotizing and perforating mesentery of the sigmoid colon (b: laparoscopy, c: open laparotomy).

**Fig. 4:**
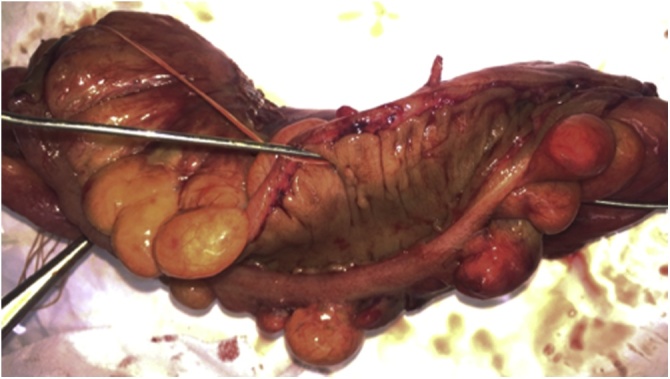
In the macroscopic examination of the surgical specimen, the diverticulum communicates with the outside of the mesentery without signs of malignancy (the inserted probe penetrated the lumen).

A 57-year-old man presented to our institution with a history of chest pain of a few days. The patient’s medical history included malignant rheumatoid arthritis while using steroids and an immunosuppressive agent. Because of interstitial pneumonia, he had been inhaling oxygen (3 L/min) at home. One year prior, the patient experienced mediastinal emphysema due to pneumothorax and was cured via nonoperative management. Vital signs were stable. Snowball crepitations were noted on the chest and neck. The abdomen was not distended, and there was no abdominal pain or sign of peritoneal irritation. A recurrence of pneumothorax was suspected, and chest radiography was performed, which revealed subcutaneous emphysema of the neck, mediastinal emphysema, and subdiaphragmatic free air (Fig. 1). Computed tomography (CT) of the neck, thorax, and abdomen was performed to rule out gastrointestinal perforation. This showed extensive retroperitoneal and mediastinal emphysema, including emphysema of the sigmoid colon mesentery (Fig. 2a–d). No evidence of pneumothorax was detected. Laboratory tests revealed increased white blood cell count (11,240/μL) and an elevated C-reactive protein level (17.85 mg/dL). A perforation in the mesentery due to sigmoid colon diverticulitis was strongly suspected. Diagnostic laparoscopy was performed under general anesthesia, and it revealed that the perforation was in the sigmoid mesentery (Fig. 3a–c). Segmental resection of the sigmoid colon and end-colostomy (Hartmann’s procedure) was performed via open laparotomy. Macroscopy of the surgical specimen revealed that the diverticulum was communicating with the outside of the mesentery without signs of malignancy, thus, it was diagnosed as a diverticular penetration (Fig. 4). The subcutaneous and mediastinal emphysema disappeared a few days after the surgery, and the patient was transferred from the intensive care unit to the general ward on the postoperative day 3.

## 3 Discussion

Colonic perforation is most often due to diverticulitis, neoplasm, and iatrogenic and traumatic mechanisms. Colonic diverticulosis is common in the developed world, affecting up to 50% of adults in Western countries [10]. Among patients with colonic diverticulosis, approximately 20% may develop diverticulitis due to infection and inflammation of the diverticula [11]. Perforation of diverticulitis is one of the most serious complications and requires an urgent operation. In some previous reports, colonic perforation resulted in a free hole occurring on the opposite side of the mesentery [12,13]. In cases of free perforation, clinical features include massive free air on CT and signs of peritoneal irritation; thus, it is easy to diagnose this early. However, spontaneous mediastinal and subcutaneous emphysema commonly occur when increased intra-alveolar pressure such as from asthma, cough, or mechanical ventilation leads to the rupture of marginal pulmonary alveoli. In particular, massive mediastinal emphysema in colonic perforation is an unusual complication, which is extremely rare without a preceding endoscopic procedure [[2], [3], [4], [5]]. To the best of our knowledge, in the English literature, only 20 cases of mediastinal emphysema following colonic perforation, excluding iatrogenic and traumatic injury, have been reported (Table 1).

**Table 1:** Mediastinal emphysema as a rare complication of colon perforation reported in the English literature.

Case	Year	Author	Age/sex	Cause	Remarks	Presenting symptom	Peritoneal irritation	Time from onset (days)	Site	Surgery	Outcome
1	1968	Maw	90/F	Diverticulitis	None	Constipation, Subcutaneous emphysema	None	3	Sigmoid colon	NOM	Survived
2	1973	Suros and Lee	80/F	Diverticulitis	NA	Abdominal pain, fever	NA	1	Sigmoid colon	colostomy	Survived
3	1973	Suros and Lee	63/M	Diverticulitis	NA	Abdominal pain, fever, shortness of breath	NA	19	Sigmoid colon	colostomy	Survived
4	1973	Dharia and Shah	66/M	Diverticulitis	NA	Substernal pain	None	1	Sigmoid colon	NOM	Survived
5	1980	Mogan et al.	22/M	Ulcerative colitis	Steroids and immunosuppressive agent	Abdominal pain, fever,	None	1	NA	Subtotal colectomy	Survived
6	1980	Mogan et al.	16/F	Ulcerative colitis	Steroids	Abdominal pain, fever	None	10	NA	Subtotal colectomy	Survived
7	1995	Hur et al.	67/F	Diverticulitis	Diabetes Mellitus	Neck swelling	None	7	Sigmoid colon	Hartmann's procedure	Survived
8	2000	von Oers et al.	66/M	Diverticulitis	NA	Abdominal pain, fever	NA	NA	Sigmoid colon	Hartmann's procedure	Survived
9	2002	Soliani et al.	63/M	Diverticulitis	Steroids	Abdominal pain	None	a few days	Sigmoid colon	Hartmann's procedure	Survived
10	2004	Becic et al.	50/F	Diverticulitis	Steroids and immunosuppressive agent	Neck swelling	None	3	Descending colon	Segmental resection and anastomosis	Survived
11	2007	Arana-Arri et al.	75/F	Stercoroma	Liver cirrhosis	Abdominal distension	NA	4	Rectum	Hartmann's procedure	Died
12	2008	Schmidt et al.	69/M	Diverticulitis	Steroids	Neck swelling, abdominal pain	NA	1	Sigmoid colon	Hartmann's procedure	Died
13	2009	Wawrzak et al.	58/M	Colon cancer	Steroids	Abdominal pain	None	2	Descending colon	Hartmann's procedure	Survived
14	2009	Wawrzak et al.	60/M	Rectal cancer	Radiation and Chemo therapy	Lower limb pain	None	14	Rectum	colostomy	Survived
15	2010	Choi et al.	59/M	Diverticulitis	None	Abdominal pain	None	30	Sigmoid colon	Hartmann's procedure	Died
16	2012	Annahazi et al.	19/M	Ulcerative colitis	Steroids and immunosuppressive agent	Chest pain, shortness of breath	None	7	NA	NOM	Survived
17	2014	Wiles et al.	81/F	Diverticulitis	Steroids and immunosuppressive agent	Abdominal pain	None	NA	Sigmoid colon	NOM	Survived
18	2014	Fosi et al.	57/M	Diverticulitis	None	Abdominal pain	None	a few days	Sigmoid colon	Hartmann's procedure	Survived
19	2015	Montori et al.	59/F	NA	None	Abdominal pain	None	NA	Descending colon	colostomy	Survived
20	2018	The present case	53/M	Diverticulitis	Steroids and immunosuppressive agent	Chest pain	None	5	Sigmoid colon	Hartmann's procedure	Survived

Abbreviations: NA, no available information NOM, non-operative management.

All cases involved perforation into the retroperitoneal or intramesenteric space. The most common cause of subcutaneous and mediastinal emphysema of colonic origin was diverticulitis (13 cases) [[2], [3], [4], [5],^7^,[14], [15], [16], [17], [18], [19]]. Our case also showed a wide range of emphysema from the mesentery to the retroperitoneum, mediastinum, and subcutaneous tissue. The pressure gradient between the gastrointestinal lumen and the retroperitoneal and subcutaneous tissue is the mechanism by which colonic perforation presents extensive subcutaneous and mediastinal emphysema. The retroperitoneal cavity is divided into three large areas and small parts; the three areas are the anterior pararenal space, the perirenal space, and the posterior pararenal space [20]. The anterior pararenal space is continuous with the mesentery, the small intestine, and the colon, and the head side of the posterior pararenal space is linked to the diaphragm, continuing to the mediastinum [[20], [21], [22]]. Quigley et al. reported that gastrointestinal tract pressure would be as high as 60 mmH_2_O in its natural state, and that gas would spread from the mesentery to the retroperitoneal space beyond the diaphragm [23]. Depending on the location of the perforation, signs of peritoneal irritation may be evident, but they can be hidden in cases of retroperitoneal colonic perforation. Colonic gas may spread via various anatomical pathways when perforation of the colon occurs in the retroperitoneum; thus, diverse atypical clinical symptoms may be present [24]. The atypical manifestation of a retroperitoneal colonic perforation can cause difficulties in making a diagnosis [[2], [3], [4], [5],^24^].

Additionally, in patients receiving steroid administration, abdominal signs are generally poor. Particularly, in patients receiving prednisone at 20 mg/day or more, clinical symptoms may not be noticeable even in cases of peritonitis resulting from a perforation and an abscess [25]. Remine et al. reported a colonic perforation due to steroid administration. They found that the mortality rate of patients who were receiving a dose of 20 mg/day or less was 13.3%, while that of those who were receiving a dose of 20 mg/day or more was 85.1% [26]. In our case, high-dose prednisolone therapy had been administered twice to treat malignant rheumatoid arthritis, and an average of 22.5 mg of prednisolone was administered. We carefully conducted a physical examination, but we did not identify any spontaneous pain in the abdomen or symptom of peritoneal irritation. In the 20 cases presented in Table 1 (including our own case), steroids and immunosuppressive agents were administered; symptoms could have been masked. Moreover, the duration from the onset of symptoms to the initiation of therapy was relatively long (Table 1); the emphysema may have widely spread.

Diagnosis in a patient with covert symptoms can be challenging for surgeons; thus, delayed diagnosis may be occurred especially in the absence of peritoneal signs and abdominal pain due to both steroid use and intramesenteric perforation. A CT scan in stable patients can be useful to detect direct evidence of colonic perforation and penetration, such as retroperitoneal and mesenteric emphysema. Finally, we suspected that the retroperitoneal and mediastinal emphysema may be from the thorax. The proportion of retroperitoneal emphysema due to intrathoracic factors was only 3.5%, which is relatively rare. In our case, judging that the mediastinal emphysema was due to the penetration of the colonic diverticulitis might be valid from two standpoints: the colonic penetration was obvious from the CT scan and there was no evidence of pneumothorax. The fact that the emphysema from the colon expanded to the mediastinum is suggestive of mediastinal infection, and delay in surgical treatment should increase the risk of developing mediastinitis. Therefore, laparotomy should be performed immediately in cases of colonic penetration of diverticulitis, where the emphysema expands to the mediastinum extensively.

## 4 Conclusions

Massive mediastinal and retroperitoneal emphysema may be rare signs of colonic perforation. Despite the presence of colonic penetration, the diagnosis may be delayed due to the absence of peritoneal irritation. Emergency laparotomy, including diagnostic laparoscopy, should be considered in cases of colonic penetration of diverticulitis, where the emphysema expands to the mediastinum extensively.

## Conflict of interest

The authors of this paper declare that they have no competing interests regarding the publication of this paper.

## Funding

This research did not receive any specific grant from funding agencies in the public, commercial, or not-for-profit sectors.

## Ethical approval

This study is exempt from ethical approval in our institution.

## Consent

Written informed consent was obtained from the patient for the publication of this case report and any accompanying images.

## Author contribution

TM drafted the article. EH, AK, KT, SK, KO, YS, and HW carried out the acquisition of data. HW participated in the critical revision of the manuscript. All authors read and approved the manuscript. TM take responsibility for the paper as a whole.

## Registration of research studies

researchregistry4660.

## Guarantor

TM take responsibility for the paper as a whole.

## Provenance and peer review

Not commissioned, externally peer-reviewed.
